# Reconstruction of finger joints using autologous rib perichondrium – an observational study at a single Centre with a median follow-up of 37 years

**DOI:** 10.1186/s12891-020-03310-5

**Published:** 2020-04-29

**Authors:** Daniel Muder, Ola Nilsson, Torbjörn Vedung

**Affiliations:** 1 Department of Surgical Sciences/Orthopedics & Hand Surgery, Uppsala University Hospital, Entrence 70 1 floor, 751 85 Uppsala, Sweden; 2 Department of Orthopedics, Falu Lasarett, Lasarettsvägen 10, 791 82 Falun, Sweden; 3 School of Medical Sciences, Örebro University and University Hospital, Örebro, Sweden; 4 Division of Pediatric Endocrinology and Center of Molecular Medicine, Department of Women’s and Children’s Health, Karolinska Institutet and University Hospital, Stockholm, Sweden; 5 Elisabeth Hospital Aleris, Geijersgatan 20, 752 26 Uppsala, Sweden

**Keywords:** Articular cartilage, Perichondrium, Transplantation, Joint reconstruction

## Abstract

**Background:**

Gratifying long-term results are difficult to achieve when reconstructing osteoarthritic finger joints. Implant surgery is the most commonly used method to restore function and dexterity. However, all types of implant have disadvantages and may be a less favorable option in some cases, especially in young patients with a long expected lifetime and high demands on manual load. Implant related complications as loosening, instability, subsidence and stiffness are the main concerns. In this context, joint reconstruction using rib perichondrium might be a reasonable alternative in selected cases. The aim of the study was to evaluate the long-term results of finger joint reconstruction using rib perichondrial transplantation.

**Methods:**

The study group (*n* = 11) consisted of eight individuals reconstructed in the proximal interphalangeal (PIP) joints and three reconstructed in the metacarpophalangeal (MCP) joints during 1974–1981. All patients were evaluated at clinical visits (median: 37 years after perichondrial transplantation, range: 34–41 years) using radiographs, disability in arm-shoulder-hand (DASH) score, Visual Analog Scale (VAS), range-of-motion (ROM) and manual strength (JAMAR).

**Results:**

None of the 11 patients had undergone additional surgery. All of the PIP-joints (*n* = 8) were almost pain-free at activity (VAS 0,6) (range 0–4), had an average range-of-motion of 41 degrees (range 5–80) and a mean DASH-score of 8,3 (range 1–51). The mean strength was 41 kg compared to 44 kg in the contralateral hand (93%). The three MCP joints were almost pain-free at activity (VAS 0,7), (range 0–1). The ROM was on average 80 degrees (range 70–90) and the mean DASH-score was 2 (range 1–3). The mean strength was 43 kg compared to 53 kg in the contralateral hand (81%).

**Conclusions:**

Perichondrium transplants restored injured PIP and MCP joints that remained essentially pain-free and mostly well-functioning without need for additional surgeries up to 41 years after the procedure. Additional studies are needed to evaluate long-term results in comparison to modern implants and to better describe the factors that determine the outcome of these procedures.

**Level of evidence:**

Level IV, Therapeutic Study.

## Background

A variety of methods aiming towards cartilage repair or regeneration have been developed and tested over the years [1, 2]. Early attempts to transplant autologous cartilage for reconstructive purposes to the ear, nose or joints failed. Some of the early investigations used autologous costal cartilage that was diced, sliced or molded into a usable structure, and then transplanted [3]. However, these experimental models did not produce new cartilage or were not applicable clinically. Eventually, pieces of cartilage were successfully transplanted into small articular cartilage defects and this strategy has been further developed and is now standard clinical practice, especially for traumatic injuries in athletes [4–6]. In addition, autologous chondrocyte transplantation is used at some centres [4, 7, 8], but this technique is not useful in small joints when the entire joint surface must be restored. Finding alternative methods required suitable tissues to transplant as well as solving the problem with attachment of the graft to the recipient site. In this context rib perichondrium was found to be a suitable tissue [9].

The perichondrium is a layer of dense connective tissue that surrounds the cartilage of the developing skeleton. It also surrounds non-skeletal cartilaginous tissues including the tip of the nose and the helix of the ear [10]. It consists of two separate layers: an outer fibrous layer important for mechanical and structural support and an inner cambium layer containing osteochondroprogenitor cells [11]. Perichondrial cells are known to play a role in regulating chondrocyte differentiation and respond to signals from underlying chondrocytes [12–14]. For example, in the cartilaginous bone templates of long bones, chondrocytes undergo proliferation, column formation and hypertrophy resulting in longitudinal bone growth. Simultaneously, flanking perichondrium differentiates into periosteum and serves as a source for both trabecular and cortical osteoblasts [15–17]. Early clinical observations suggested and experimental studies supported that perichondrium also has chondrogenic potential [18–20]. This chondrogenic potential of perichondrium and its potential for regeneration of articular cartilage was further investigated in the 1970s [3, 9] and has since then been used at our centre to reconstruct articular surfaces in small joints damaged by infection and/or trauma [21, 22]. It was a relatively widespread surgical technique during the following decades [23–25]. However, starting in the early 90s, partly due to variable results [22, 24] and partly due to improved implants, the usage of the technique declined. The need of a second surgical site may also in part explain the limited use of this method [26]. The aim of the present study was to evaluate the long-term outcome after all perichondrial transplantations to the proximal inter-phalangeal (PIP) joint and the metacarpophalangeal (MCP) joint of the hand performed at Uppsala University Hospital during 1974 to 1981.

## Methods

### Retrospective chart review of perichondrial transplantations to the PIP and MCP joints

In order to evaluate long-term clinical outcome of joint reconstruction by perichondrial transplantation, a retrospective chart review was performed and identified 14 living and locatable patients that had undergone autologous perichondrial transplantation to the PIP and MCP joints between 1974 and 1981 at Uppsala University Hospital. The timeframe starts with the primary case in 1974 and ends in 1981 when the journal system was altered. Twenty-six patients could not be included in the study due to the following reasons: 19 deceased, 7 not located. Hence, the loss to follow-up was 26/40 (65%). In addition, three of the PIP cases were excluded since they had been converted into fusion shortly after the primary surgery (range 3–25 months postoperatively) and had no joint to asses. The reason for these failures was persistent pain and stiffness. The remaining and final study group (*n* = 11) included 8 patients with reconstructed PIP joints and 3 patients with reconstructed MCP joints. They were contacted by letter and all responded and agreed to participate in the study by written informed consent.

The patients were assessed with plain radiographs and by measuring range-of-motion (ROM) with goniometer. Manual strength was assessed by a JAMAR hand dynamometer (Patterson Medical Ltd., Nottinghamshire, UK). Pain was assessed with Visual Analog Scale (VAS), (scale: 0 (no pain) to 10 (most severe pain)). Manual ability was assessed with Quick DASH (The disabilities of the arm, shoulder and hand score), (scale: 0 (no disability) to 100 (most severe disability) [27]. All measurements and examinations were done by the same observer (DM).

In all cases but one the joint problem was caused by dislocation, intra-articular fracture and/or posttraumatic infection. The time from injury to surgery was on average about 10 months (range 1–33 months) (Tab. 1). Several of the subjects have been active in physically demanding occupations for many years after the surgery.

**Table 1:** Indications for surgery and long-term clinical outcome after joint reconstruction

Case	Sex (M/F)	Joint	Year of surgery	Injury type / Cause	Main symptom	Time to surgery (months)	Age at surgery (years)	ROM (degrees At early follow-up times according to medical charts	Follow-up (years)	ROM (degrees)	Extension deficit (degrees)	VAS rest	VAS act.	DASH score	JAMAR Op side (kg)	JAMAR Non-op side (kg)	Strength op vs Non-op side (in percent)
1	F	PIP IV sin	1976	Idiopathic osteoarthritis	Pain	–	52	0–80 10 years post-op	39	80	0	0	1	51.7	7	9	78
2	M	PIP IV dx	1977	Fracture dislocation	Pain	12	22	Not available	38	5	25	0	0	6.7	45	57	79
3	F	PIP II dx	1977	Closed dislocation	Pain	4	20	10–80 6 months post-op	38	70	0	0	0	3.4	26	30	87
4	F	PIP III dx	1978	Open fracture, infection	Stiffness	6	20	25–60 6 months post-op	37	10	10	0	0	2.5	29	30	97
5	M	PIP IV dx	1978	Intra articular fracture	Pain	16	22	30–55 6 months post-op	37	20	30	0	0	1.7	38	39	97
6	M	PIP III dx	1978	Intra articular fracture	Pain, Stiffness	11	16	Not available	37	80	0	0	0	2.5	77	70	110
7	M	PIP II dx	1980	Saw injury, fracture	Stiffness	7	31	40–80 6 months post-op	35	20	10	0	0	0	54	53	102
8	M	PIP III dx	1981	Closed PIP dislocation	Pain	33	26	5–60 9 months post-op	34	40	5	0	4	0.8	52	62	84
9	M	MCP III dx	1974	Saw injury, infection	Pain, Stiffness	8	20	5–80 41 months post-op	41	75	5	0	0	0	53	63	84
10	M	MCP IV sin	1978	Shotgun injury	Stiffness	2	12	Not available	37	70	0	0	0	4.2	36	54	67
11	M	MCP III sin	1979	Fight Bite, infection	Pain	1	24	Not available	36	90	0	0	1	3.3	40	42	95

**Fig. 1:**
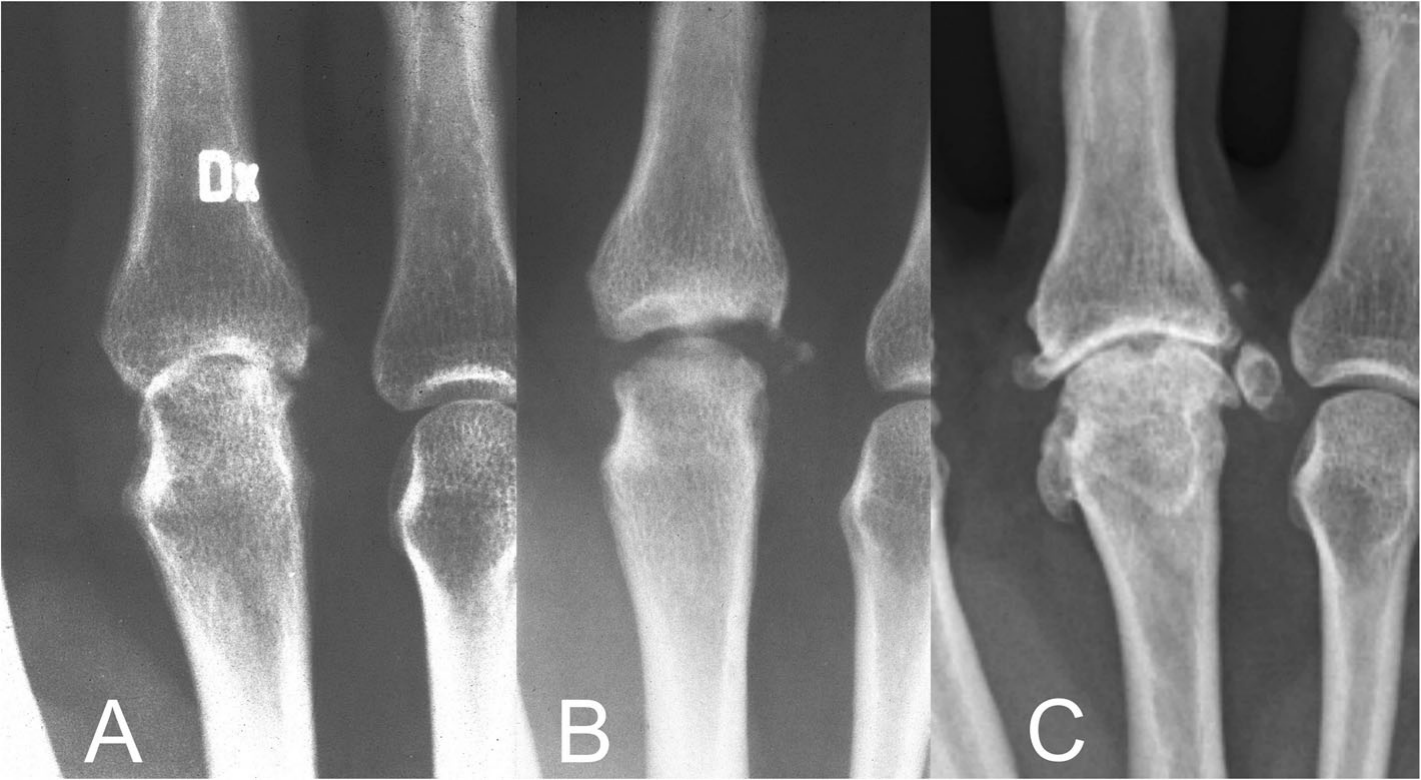
Radiographs (AP view) of the third metacarpophalangeal joint in the primary case from 1974 (case 9) before (**a**), 6 months after (**b**), and 40 years after (**c**) perichondrial reconstruction of the joint. Reproduced with permission of SAGE Publishing [28]

**Fig. 2:**
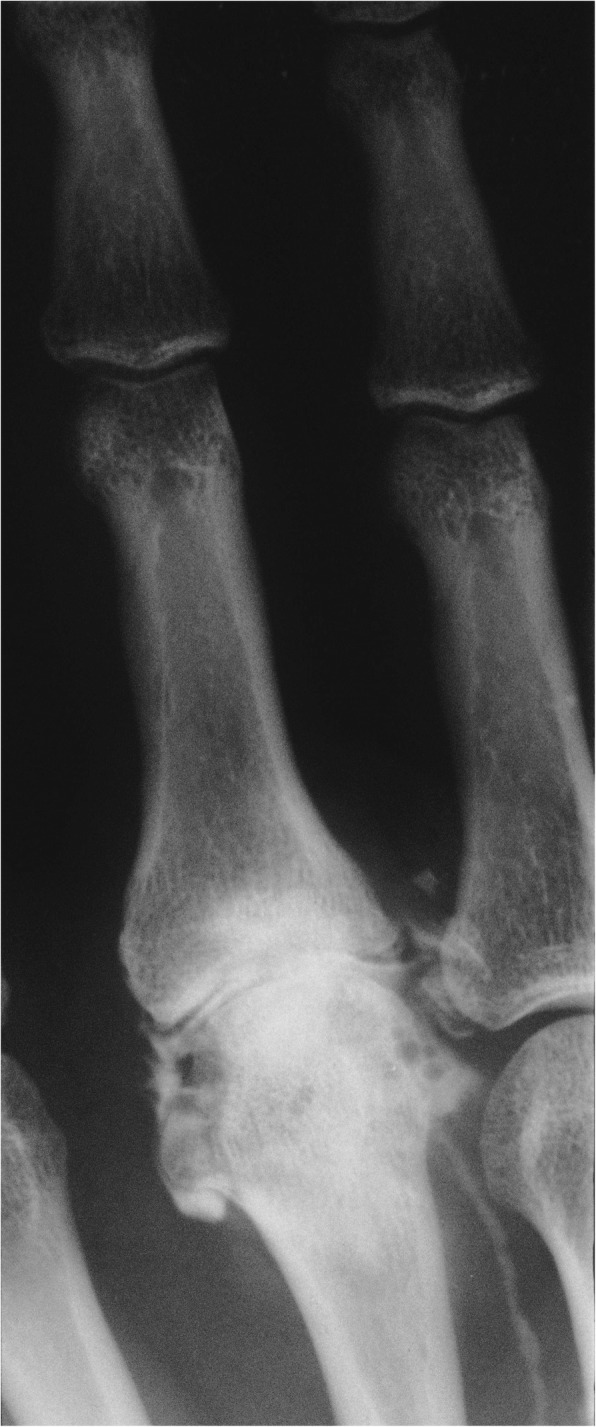
Arthrography 3.5 years postoperatively, revealing some peri-articular irregularities but a smooth joint space with even surfaces. Some contrast is also seen proximally in an inter-metacarpal lymph vessel. Reproduced with permission of SAGE Publishing [28]

Some of the cases in the present cohort has been reported previously at early stages after the surgery including the first case from 1974. In the primary case preoperative radiographs revealed severe osteoarthritis with destruction of the articular cartilage (Fig. 1a; reproduced with permission of SAGE Publishing) [28]. Radiographs, 6 months postoperatively, showed a wider joint space compared to the preoperative findings and some slight irregularities in the subchondral bone (Fig. 1b; reproduced with permission of SAGE Publishing) [28]. An arthrography 3.5 years postoperatively indicated smooth and congruent joint surfaces (Fig. 2; reproduced with permission of SAGE Publishing) [28],

### Surgical technique

Standardized surgical technique in brief, all remnants of the eroded joint surfaces (both sides of the joint) are resected down to bleeding subchondral cortex. Care is taken to preserve the shape of the joint surfaces. The perichondrium is harvested from the 6th or 7th rib. A skin incision is made in the sub-mammary crease, starting from the osteochondral junction (a slightly elevated rim in the mid-clavicular line) and stopped at the medial margin of the crease to avoid unsightly scarring. The rectus abdominis fascia and underlying muscle is divided transversely to expose the donor site. The perichondrium is incised along the superior and inferior borders of the cartilaginous part of the donor rib. A transverse incision is made in the rib periosteum a few millimetres lateral to the bone-cartilage rim. This small portion of periosteum is temporarily included in the graft to enable grasping and pulling. The perichondrium is lifted at the bone-cartilage rim of the rib and peeled off the underlying cartilage with a blunt dissector all the way to the sternum. The last centimetres of dissection from medial margin of the skin incision to the lateral margin of the sternum is done subcutaneously. Care is taken not to include any cartilage and not to damage the cambium (inner) layer of the perichondrium. The perichondrium is osteo-sutured at the joint margins of the recipient site with the cambium layer facing towards the joint space and the outer fibrous layer facing the subchondral bone. A thin silicone sheet is temporarily placed in the joint and helps mold the transplant and prevents adherence between the reconstructed joint surfaces. The hand is immobilized in a cast in “the position of safety” [29] for 4 weeks, followed by physiotherapy. The silicone membrane is subsequently removed in a second operation, after 4 months in the present study group. In the beginning of the 80’s the technique was slightly modified as fibrin glue was added to reinforce the attachment of the graft [30]. The cohort in the present study was operated before this modification.

### Statistical methods

The difference in grip strength between operated and unoperated joint/hand were analyzed with paired t-tests and data were expressed as mean ± SD. All data were analysed using the SPSS software version 23 (IBM).

## Results

### Clinical assessment

All patients (*n* = 11) underwent clinical evaluations including assessment of pain, range-of-motion (ROM), and function (DASH) on average 37 years (range 34–41 years) after the original procedure. None of the 11 patients had received additional surgical treatment of the reconstructed joints. The eight PIP joints were pain-free at rest (average VAS 0; Tab. 1) and almost pain-free during activity (average VAS 0.6: range 0–4; Tab. 1). The total range-of motion (ROM) was on average 41 degrees (range 5–80 degrees; Tab. 1). Three of the PIP joints had nearly full ROM (70–80 degrees). In three PIP joints the ROM were 20–40 degrees, which was less than optimal but still allowed for a reasonable function in the affected fingers. The remaining two PIP joints had poor ROM (only 5–10 degrees). The mean DASH-score was 8.7 (range 0–51; Tab. 1). The DASH score was 6.7 or lower in all cases but one. The high DASH-score in case 1 is probably influenced by other problems as indicated by the impaired manual strength on both sides and the age of the subject (91 years). Average hand strength was similar in the operated hand compared to the contralateral hand (41 kg ± 21 vs. 44 kg ± 20; *P* = 0.24; Tab. 1). The mean extension deficit was 10 degrees (range 0–30).

**Fig. 3:**
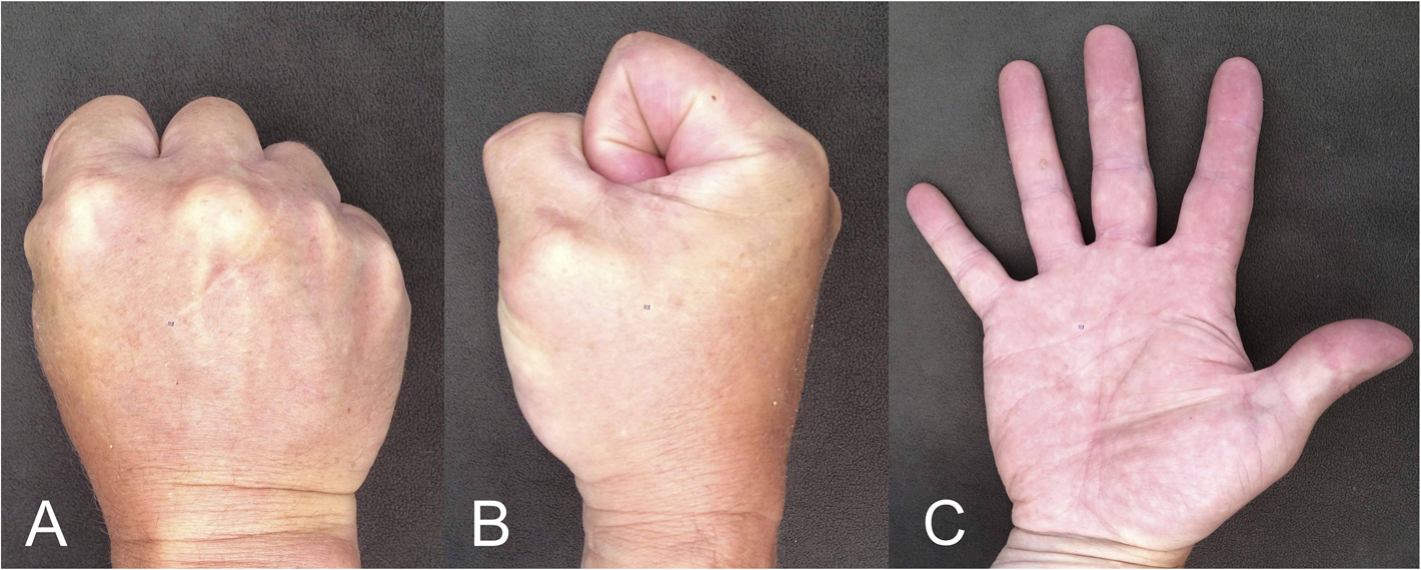
The range-of-motion in the reconstructed third metacarpophalangeal joint in the primary case from 1974, 40 years after the surgery: **a**, Flexion (dorsal view); **b**, flexion (lateral view); **c**, extension (volar view)

The three MCP joints were pain-free both at rest (average VAS 0) and during activity (average VAS 0; Tab. 1). Five degrees’ extension deficit was found in one out of three cases, but the total ROM was essentially normal in all three patients (average 78 degrees; range 70–90) (Fig. 3). The mean DASH-score was 2.5 (range 1–3). The mean strength was similar in the operated hand compared to the contralateral hand (43 kg ± 8.9 vs. 53 kg ± 10.5; *P* = 0,16; Tab. 1).

None of the patients had any donor site morbidity or complaints. The resulting scars were effectively hidden in the sub-mammary crease in all available patients (*n* = 11).

### Radiological assessment

**Fig. 4:**
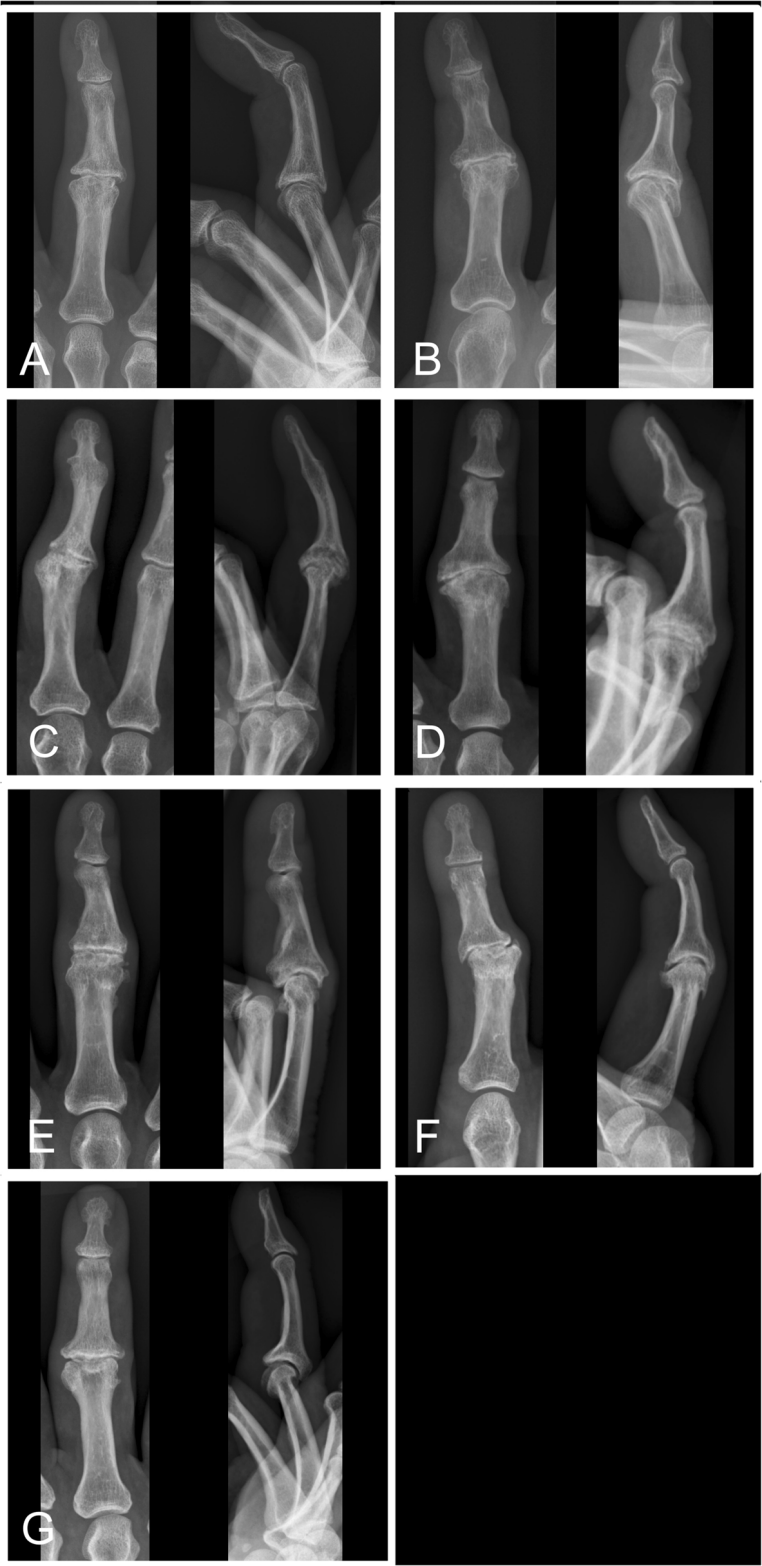
Radiographs of the reconstructed proximal interphalangeal joints in case 2–8 (**a**-**g**). No radiographs were obtained in case 1 before the patient passed away

**Fig. 5:**
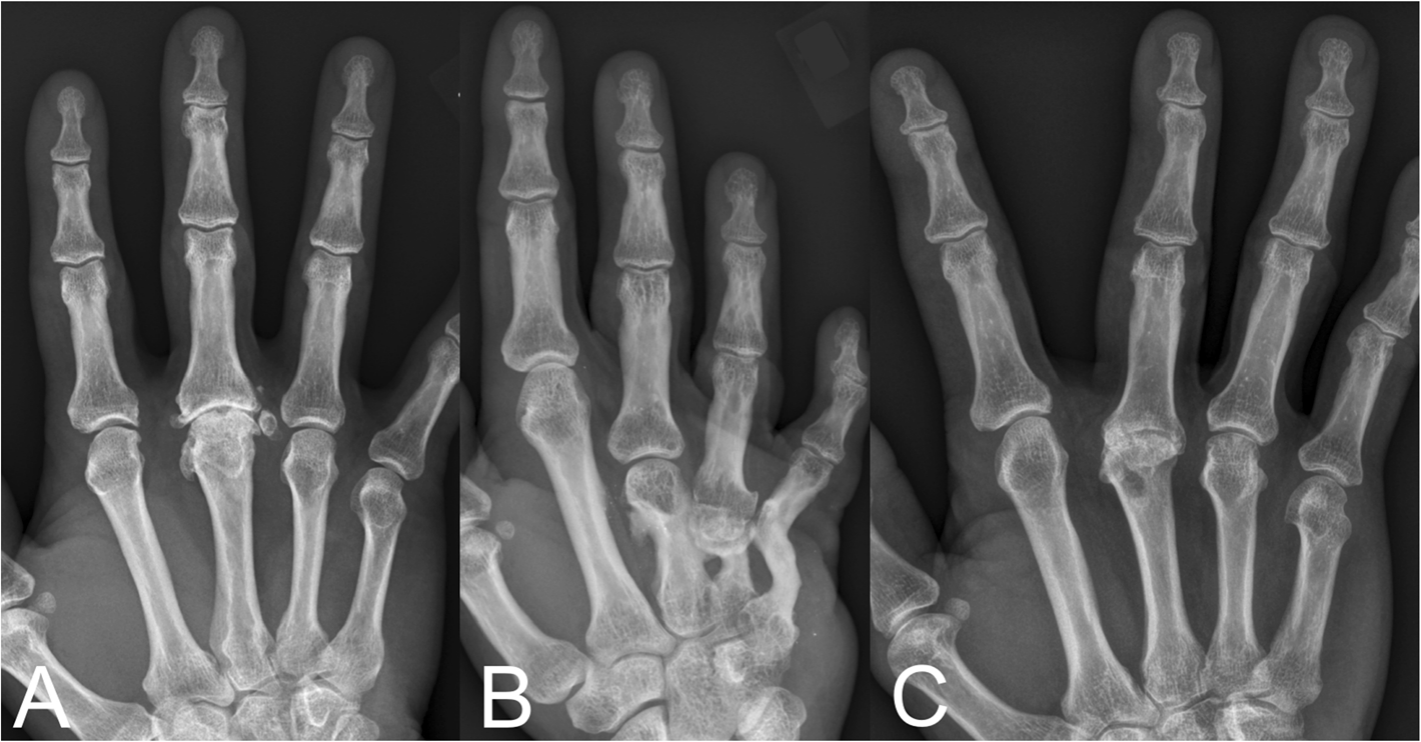
Radiographs of the reconstructed metacarpophalangeal joints in case 9–11 (**a-c**). The joint space in case 10 (MCP IV) was difficult to visualize due to the bony deformities caused by the shotgun injury in 1978

Radiographs of the primary case obtained before (Fig. 1a) and 6 months after the procedure (Fig. 1b) indicate that a wider joint space is regained after the surgery. Arthrography 3.5 years postoperatively, suggested smooth joint space with even surfaces and only minor irregularities. However, at long time follow-up (average follow-up time 37 years) radiographs in several cases showed significant signs of osteoarthritis including subchondral sclerosis, cysts, loose bodies and osteophytes at the joint margins (Fig. 1c, Fig. 4, Fig. 5). Interestingly, radiographic osteoarthritis did not correlate with clinical signs of osteoarthritis. This discrepancy between clinical impression and radiological appearance has also been reported at short term follow-up studies [26, 28].

## Discussion

The main goal of the present study was to assess long-term outcome after perichondrial transplantation to the PIP and MCP joints of the hand. We found that none of the 11 cases in the study cohort had required any second surgery, that all remained reasonably well functioning and pain-free at rest, and that all patients but one, were essentially pain-free during exercise and strain (Tab. 1).

Perichondrial transplantation has been applied to a variety of joints; e.g. different joints in the hand, the wrist, the elbow, the knee, and the foot [22, 23, 25, 31]. Minor modifications of the original surgical technique and the postoperative regime was introduced in the 80’s; in addition to osteo-suture, attachment of the graft is often reinforced with fibrin glue [30], and extraction of the silicone membrane is nowadays normally done after 6–8 weeks [26].

However, there are few long-term outcome reports in the literature. In 1980, Engkvist et al. reported short-term results (range 3–41 months) of twenty-six perichondrial transplantations [22]. The surgical indication varied considerable in the study group, e.g. congenital malformations, rheumatoid arthritis, degenerative osteoarthritis, and post-traumatic osteoarthritis that in some cases were associated with significant soft tissue trauma. The operated joint also varied significantly; eleven MCP joints, eight PIP joints, four trapezio-metacarpal joints, two metatarsophalangeal joints of the big toe and one elbow. The interpretation of the results in such variable cohort becomes difficult and the outcome was, as could be expected, variable but in some cases, excellent [22]. Seradge et al. reported a retrospective study of thirty-six perichondrial transplantation with a minimum follow-up of three-years [24]. This study group was more homogenous with sixteen MCP joints and twenty PIP joints. The results graded as good (no pain, absent or occasional swelling, and useful ROM) were comparable in the MCP (56%) and the PIP joints (55%). They concluded that the outcome was better in younger patients and suggested that the method should be avoided in patients older than 40 years of age. Although there was a tendency towards better results in teenagers and young adults, this conclusion seems to be based on very few observations. Only two patients older than 40 years of age were available for age evaluation (one MCP and one PIP joint) and both were graded as fair (useful ROM with or without minimal swelling and occasional discomfort) [24] and the negative effect of increasing age may thus be over-stated in this study.

In our series, only one patient was older than 40 years (52 years) at the time of surgery (Tab. 1, case 1). However, in this patient the reconstructed PIP joint had excellent ROM (80 degrees) with minimal pain (VAS 1) at activity 39 years later, demonstrating that favourable long-term out-comes is possible beyond young adulthood.

The discrepancy between the clinical impression and the radiological appearance is interesting. The irregularities were mostly seen in the joint margins as osteophytes (Fig. 4-5). The joint space in the reconstructed joints was in general narrower than in the neighbouring normal joints but the joint surfaces seem to be smooth. Resorption and remodelling of the cortical bone contour have been reported to occur after perichondrial transplantation [26, 28], and similar finding were found in the present study.

The total ROM in the primary case from 1974 was 75 degrees (range 5/80) 41 months after the surgery [22], which is similar to our findings during the 40-year follow-up evaluation (Tab. 1, case 9, Fig. 3). The relative grip strength in the operated and un-operated (dominant) hand has remained similar over time, 95% (95/100 kg) in the 41-months follow-up and 84% in the 40-year follow-up (53/63 kg). The reduction in strength is similar to the decline in strength that occurs with age in the general population [32]. The ROM at various early follow-up times in seven of the cases are displayed in Table 1. In five of the cases (case 1,3,5,8–9) the short-term results are almost identical to the long-term results obtained several decades later (Tab. 1). In the 70’s, available implants consisted mainly of silicone prostheses [33]. However, even if a modern non-constrained implant may have restored the function equally well, it would probably not have lasted for 35–40 years without the need for a re-surgery. In addition, revision surgery after a failed implant is difficult due to the altered anatomy. In contrast, with the perichondrial transplantation technique, the option for later implant arthroplasty is preserved since most of the bone and soft tissues around the joint is left intact. Additional studies are required to develop and evaluate the current method. New experimental techniques and approaches including cell-tracing technologies have opened new possibilities to study skeletal development, growth, and regeneration [34] and may thus be helpful to evaluate the cellular origin and mechanisms important for the outcome of the current surgical method.

The extensive observation period is both a strength and a weakness. It enables a rare opportunity to asses clinical outcome several decades after the surgery. It is also coupled with an inevitable and large loss to clinical follow-up. All patients who fulfilled the inclusion criteria; perichondrium transplantation to the PIP or MCP joints, being locatable and alive, responded and agreed to participate in the study. Three of the PIP joints identified in the retrospective chart review had been converted to fusion shortly after the primary surgery and were excluded in the present study, as they had no joint to assess. However they are important to display and consider. The postoperative complications that necessitated these fusions included persistent pain and stiffness, but the mechanistic reasons for these failures could not be elucidated from the patient files. Potential causes may be poor transplant quality or detachment of grafts. In addition, this cohort of patients was the first to undergo perichondrial transplantation, a completely new method at the time. The number of cases in the present study is relatively small, especially in the MCP group, which limits interpretation of the results. There were only three MCP cases and the positive results in this study may be reproducible in future studies with larger number of patients. Another possibility for the favourable results may be that articular cartilage in some cases was not completely destroyed. Remnants of native cartilage could potentially have contributed to the favourable outcomes. However, this is unlikely since the arthritic joint surfaces were almost completely devoid of articular cartilage before the procedure and then resected and completely covered with perichondrium during the procedure [21, 35]. Furthermore, due to the limited regenerative capability of articular cartilage, it is less likely that small pieces of cartilage remnants would have made major contributions to the resurfacing of the joint surface [21, 35]. We did not have a suitable control group and it is therefore not possible to determine if transplantation of other tissues, i.e. periosteum [36] or extensor retinaculum [37], would have produce similar results.

Non-constrained surface replacement implants, with a proximal and a distal component, have become increasingly more popular during the last decades. However, regardless of type, these implants struggle with complications such as loosening, subsidence, joint instability, joint contracture, swan neck deformity, malalignment, dislocation etc. [38]. Short-term results may be gratifying but in the long-term complications often emerge (summarized in Tab. 2 [40, 42–48]). Silicone implant arthroplasty is still a widely used method to reconstruct destructed finger joints in the rheumatoid patient, especially at the MCP level [38]. However, silicone implants are a suboptimal alternative in the non-rheumatoid patients, especially in young individuals, since these implants are coupled with a high fracture rate [39, 41] (Tab. 2). In a long-term follow-up (14 y) of 52 rheumatoid patients who underwent simultaneous silicone MCP joint arthroplasties of all four fingers, implant fracture rate was as high as 63% (130/208 implants), and persistent pain ranging from occasional to constant was reported in up to 73% of the hands [39] (Tab. 2). A variety of other methods have been used to reconstruct and resurface eroded PIP and MCP joints, e.g. periosteum [36], extensor retinaculum [37] and corium (dermis graft) [50]. None of these autograft methods have been shown to be superior and they are not widely used at present. Autologous hemi-hamate autograft is another method to consider when the destructed joint surface is limited to the palmar lip of the middle phalanx after a dorsal fracture dislocation at PIP level. In a systematic review, this technically challenging method was reported to have an overall complication rate at around 35% and a long-term osteoarthritis rate as high as 50% [49] (Tab. 2).

**Table 2:** Alternative methods and reported outcome

Author	Study design	Joint	Implant	No. of Joints/ Patients	Mean age at surgery (years)	Follow-up (years)	ROM (pre-postop)	Pain	Cause for surgery (No. of Joints)	Out-come	Conclusion And Summary
**Goldfarb 2003[**39**]**	Case Series	MCP	Silicon	208/36	52	14	30° to 36°	27% pain-free	RA (208)	7% revisions 63% implant fracture	High rate of implant fractures
**Chan 2013[**40**]**	Systematic Review	PIP	Silicon Pyrocarbon	1430/x 452/x	53 58	0.5–23 1.1–5	29° to 37° 37° to 45°	76% pain-free 64% pain-free	Posttraumatic (663) RA (406) OA (193) Others (65) Posttraumatic (30) RA (22) OA (158)	4% revision 2% salvage surgery 14% revision 8% salvage surgery	High number of joints Differences in study design and parameters make comparisons difficult. Revision and salvage rates almost 4 times higher in the pyrocarbon group
**Boe 2018[**41**]**	Case Series	MCP	Silicone	325/113	64	7.2	33° to 43°	94% none or only mild pain	RA (309) OA (11) Posttraumatic (5)	7% revision 37% implant fracture in whole cohort 32% implant fracture at 10y 65% implant fracture at 15y	Progressive risk of implant fracture over time Implant fracture had no bearing on clinical outcomes
**Cook 1999[**42**]**	Case Series	MCP	Pyrocarbon	151/53 71/26 available for follow-up	58	11.7	39° to 52°	Not available	RA (62) Posttraumatic (4) others (3)	12% revision 70% 16 years survival	High loss to follow-up (53% of the patients)
**Sweets 2011[**43**]**	Case Series	PIP	Pyrocarbon	31/17	64	4.6	X° to 31° (0–100)	Average VAS 3 (0–7)	OA (31)	19% revision 48% loosening 16% dislocation	High follow-up (100%) In total 75% revision, loosening or dislocation
**Pritsch 2011[**44**]**	Case Series	PIP	Pyrocarbon CoCr-UHMWPE	203/x 91/x	51 (at revision, the whole study cohort)	Clinical data in 48 of 76 reop cases were reviewed on average 2.3y after last reop.	32° to 33° (In the follow-up cohort, *n* = 48, before first reoperation)	39% (30/76) of the patients in the reoperation cohort reported no pain	(76 reoperations in 59 patients) OA (35) Trauma (24) Inflammatory arthritis (17)	50 reoperations 26 reoperations	Mean time to first reoperation less than 1y. No significant change in preop vs postop ROM (reoperation cohort) Most patients (reoperation cohort) had mild or no pain
**Wagner 2018[**45**]**	Case Series	PIP	Pyrocarbon	170/99	Not available	6	Not available	Not available	RA (49) Trauma (29) OA (92)	34% reoperations including 21% implant revision	1 in 5 will require revision by 5y 1 in 3 will have progressive loosening or subsidence by 5y. The results are particularly concerning regarding young patients and those with posttraumatic OA
**Mora 2020[**46**]**	Case Series	PIP	Pyrocarbon	29/19	Not available	6.4	X° to 60°	VAS 1.6	Not available	24% revision	24% revision rate at mid-term follow-up 14% implant removal after 4.6y
**Murray 2012[**47**]**	Case Series	PIP	CoCr-UHMWPE	67/47	63.5	8.8	X° to 40°	VAS 3 (of 100)	OA (50) RA (17)	12% implant failure 14 of the 31 patients that returned for clinical follow-up had complications. (4 fusions, 2 amputations)	Low pain level Higher risk for implant failure/complications in RA patients.
**Jennings 2015** **[**48**]**	Case Series	PIP	CoCr-UHMWPE	39/21	62	9.3	58° to 56°	82% less pain 18% worse pain	OA (36) RA (2) Trauma (1)	26% revisions	Satisfaction rating 26/39 (67%) very satisfied
**Frueh 2015[**49**]**	Systematic Review	PIP	Hemi-hamate autograft	71 joints	Not available	3	77°	Not available	PIP fractures (71) (acute and chronic)	35% complications, 50% postop OA	High rate of postoperative OA (up to 50%)

*RA* Rheumatoid arthritis

OA Osteo arthritis

*CoCr* Cobalt Chrome

*UHMWPE* Ultra-high-molecular-weight polyethylene

The ultimate goal of an articular cartilage repair is to restore the native tissue structure but the outcome, regardless of the surgical method used, is generally believed to be fibro-cartilaginous at best [51]. The nature of the resulting tissue is scientifically interesting and important but the primary measure of success in a clinical perspective is to achieve long-term joint function and pain relief [52].

## Conclusions

In summary, we found that resurfacing of injured finger joints using transplanted perichondrium can provide acceptable long-term results. In this context, the three early failures are important to consider. However, the remaining study cohort (*n* = 11) had no additional surgery to the reconstructed joint and was assessed after an average of 37 years. Our findings suggest that function of the resurfaced joints will remain favourable in the long-term in most patients with favourable short-term outcome. Further studies are needed to determine if this method can be developed into a method that is safe and efficacious. These studies should focus on factors that may improve short-term outcomes, evaluate the surgical method in comparison to modern implant surgery, and to clarify the mechanisms by which perichondrial transplants support the formation of functional joint surfaces.

## Data Availability

The data set supporting the conclusion of this article is available on request to the corresponding author.

## Abbreviations

PIP: Proximal interphalangeal
MCP: Metacarpophalangeal
DASH-score: Disabilities of the arm, shoulder and hand score
n: Number
Kg: Kilogram
ROM: Range of motion
VAS: Visual analogue scale
IBM: International business machines corporation
Tab: Table
Fig: Fig.
RA: Rheumatoid arthritis
OA: Osteoarthritis
CoCr: Cobalt Chrome
UHMWPE: Ultra-high molecular-weight polyethylene
